# Synthesis of quasi-free-standing bilayer graphene nanoribbons on SiC surfaces

**DOI:** 10.1038/ncomms8632

**Published:** 2015-07-09

**Authors:** Myriano H. Oliveira, Jr., Joao Marcelo J. Lopes, Timo Schumann, Lauren A. Galves, Manfred Ramsteiner, Katja Berlin, Achim Trampert, Henning Riechert

**Affiliations:** 1 Paul-Drude-Institut für Festkörperelektronik, Hausvogteiplatz 5-7, Berlin 10117, Germany

## Abstract

Scaling graphene down to nanoribbons is a promising route for the implementation of this material into devices. Quantum confinement of charge carriers in such nanostructures, combined with the electric field-induced break of symmetry in AB-stacked bilayer graphene, leads to a band gap wider than that obtained solely by this symmetry breaking. Consequently, the possibility of fabricating AB-stacked bilayer graphene nanoribbons with high precision is very attractive for the purposes of applied and basic science. Here we show a method, which includes a straightforward air annealing, for the preparation of quasi-free-standing AB-bilayer nanoribbons with different widths on SiC(0001). Furthermore, the experiments reveal that the degree of disorder at the edges increases with the width, indicating that the narrower nanoribbons are more ordered in their edge termination. In general, the reported approach is a viable route towards the large-scale fabrication of bilayer graphene nanostructures with tailored dimensions and properties for specific applications.

## Abstract (web summary)

Bilayer graphene nanoribbons are very promising for future nanoelectronics. Here Lopes *et al.* show a novel approach for the fabrication of quasi-free-standing bilayer graphene nanoribbons on SiC, based on the precise control of the layer-by-layer growth of graphene and a simple annealing step in air.

## Graphical abstract

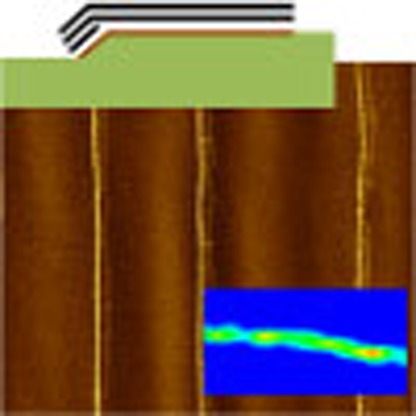

The graphene quasi-one-dimensional nanostructure called a graphene nanoribbon (GNR) has attracted much attention owing to its distinct electronic properties, which are strongly dependent on the type of edge (that is, zigzag or armchair) and the width of the ribbon^1^,^2^. Monolayer GNRs have certainly been the most investigated so far. Nevertheless, bilayer GNRs are also of special interest, as they are expected to combine the structurally and dimensionally dependent electronic properties of a GNR^3^, with the unusual features of a bilayer graphene, including its electric field tunable band-gap^4^,^5^,^6^,^7^,^8^,^9^. It has been shown that the use of bilayer GNRs in electronic devices can indeed lead to improved properties such as lower electrical noise and higher *I*_on_/*I*_off_ ratios compared with monolayer GNR counterparts^8^,^10^. Moreover, these nanostructures also offer great potential of application in advanced optics. For instance, very recent findings from Yan *et al.*^11^ reveal a novel phonon-induced transparency phenomenon in bilayer GNRs, which is associated to the peculiar plasmonic properties of bilayer graphene^12^.

Therefore, beyond the conventionally used substrate transfer and patterning of graphene sheets—two technological steps that usually result in structural damage—the direct synthesis of bilayer GNRs on an insulating template, by employing a method that allows exerting control over dimensions and edge structures, would be extremely advantageous for fundamental investigations of the pristine properties of these nanostructures, as well as for applications. In that respect, the epitaxial growth of GNRs on vicinal step facets of SiC(0001) appears as a very promising route^13^,^14^,^15^. The recently demonstrated ballistic transport in 40 nm wide monolayer GNRs attests the potential offered by these nanostructures for future graphene-based nanoelectronics^15^. So far, the emphasis has been put on the formation of monolayer-thick GNRs on SiC^14^,^15^,^16^. It is actually unlikely that the reported approaches can be generalized for the preparation of isolated bilayer GNRs on SiC. This is because the layer-by-layer nature of graphene growth on SiC(0001) (see discussion) will probably lead to the formation of a continuous monolayer graphene at the (0001) terraces, which will electrically connect bilayer graphene GNRs formed at the step facets^17^,^18^.

Here we demonstrate a novel approach for the synthesis of isolated bilayer GNRs on SiC(0001) surfaces. It is based on the precise control of the layer-by-layer growth of graphene and a straightforward annealing step in air. With this method it is possible to prepare μm-long bilayer GNRs of different widths (ranging from about 30 to 320 nm) exclusively over step edge regions of a SiC surface. The present results open a new perspective on the tailored fabrication of bilayer graphene nanostructures for different applications in nanoelectronics.

## Results

### Synthesis of bilayer GNRs

**Figure 1:**
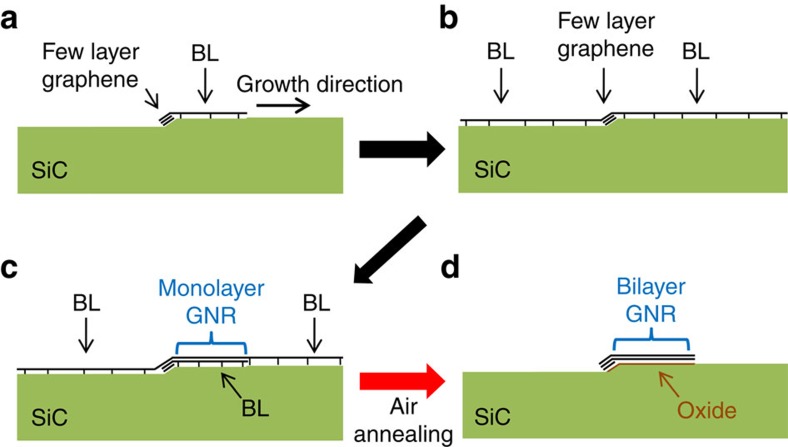
Process for the synthesis of quasi-free-standing bilayer GNRs on SiC(0001). (**a**–**c**) Different stages of the layer-by-layer graphene formation initiating on a surface step of a SiC(0001) surface. (**a**,**b**) Evolution of the growth of the (6√3 × 6√3)*R*30° BL over the SiC(0001). (**c**) Monolayer GNRs can be formed at the step edge region by controlling the growth process. (**d**) Changes promoted by air annealing: decoupling of the BL underneath the monolayer GNR from the substrate due to the oxidation of the SiC surface and etching of the bare BL. These two effects lead to the formation of quasi-free-standing bilayer GNRs exclusively at step edge regions. Note that the illustrations are not to scale.

The steps involved in the preparation of bilayer GNRs on SiC(0001) are depicted in Fig. 1. In the specific case of the SiC(0001) face, surface step edges, which can be developed by a H-etching procedure, act as nucleation centres for the graphene formation. The proper tailoring of the SiC surface morphology hinders step bunching during graphene formation and thus the modification of the starting surface^19^. Initially, Si atoms sublimate from the step edge region leaving behind a carbon-rich surface area, which propagates along the upper terraces as Si atoms further sublimate from the retracting atomic steps (Fig. 1a). This process will continue until the one-atom thick carbon-rich front reaches the next step edge (Fig. 1b), with a continuous layer finally being formed^18^,^20^,^21^,^22^. This first carbon layer is the so-called buffer layer (BL), which has a (6√3 × 6√3)*R*30° periodicity with structure and lattice parameter very similar to those of graphene. It differs from a graphene layer as it is partially bound to the SiC surface through covalent bonding^23^,^24^,^25^. It is worth noticing that already during the initial stage of the BL growth along the upper terrace, few-layer graphene can form on the vicinal facet^26^. The second layer also grows along the upper terraces below the BL (Fig. 1c), leading to the detachment of the latter from the substrate. In this way, the underlying layer becomes the new BL, while the former is converted into monolayer graphene. Consequently, if the growth process is quenched in the beginning of the formation of the second layer (Fig. 1c), the creation of monolayer GNRs (on top of the BL) at the surface terrace close to the step edge region can be realized. The variation of growth temperature will allow for the modulation of the GNR width. This happens because the temperature directly affects the sublimation of Si atoms from the surface, which in turn is directly associated with the lateral growth rate of the layers. Alternatively, the growth time can be varied for obtaining GNRs of different widths as well. Interestingly, the topmost layer of the GNR and the bare BL next to it, on the surface terraces, are formed by the same carbon layer (Fig. 1c). By allowing the growth to proceed further, a continuous monolayer graphene/BL coverage shall be formed over the whole SiC surface (not shown)^17^,^18^,^21^.

After exerting control over the layered graphene growth, the second step that allows the creation of bilayer GNRs consists of a simple annealing in air. We have previously shown that high-quality bilayer graphene can be produced by thermally treating monolayer graphene on SiC(0001) in air^27^. The BL decoupling occurs due to the formation of an oxide layer between it and the SiC surface. Very recent data reveal that by performing a standard wet oxidation annealing, the same result can be obtained^28^. A remarkable effect takes place when this same simple procedure is applied to a sample containing only monolayer GNRs at the surface (see Fig. 1c): the air annealing promotes the etching of the bare BL existing on top of the surface terraces, while the same layer existing underneath the monolayer GNRs persists throughout the process and is decoupled from the substrate (Fig. 1d). This finally leads to the creation of isolated and quasi-free-standing bilayer GNRs.

### Microscopic characterization

**Figure 2:**
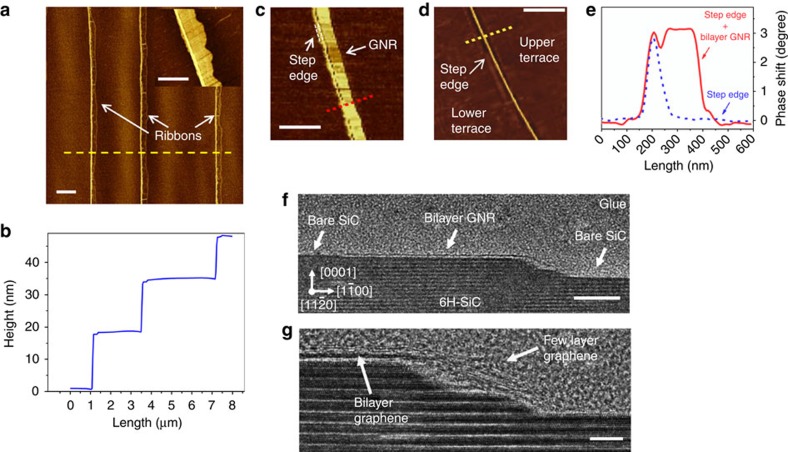
AFM and TEM results for bilayer GNRs. (**a**) AFM phase contrast image taken from a sample containing 320 nm wide bilayer GNRs (growth at 1,500 °C for 15 min followed by air annealing). Scale bar, 1 μm. The inset shows a close-up view of a single GNR. Scale bar, 0.5 μm. (**b**) Height profile taken from the same area (yellow dashed line in (**a**)). (**c**) Phase contrast image taken from a single bilayer GNR (growth at 1,470 °C for 15 min followed by air annealing). Scale bar, 0.5 μm. (**d**) Phase contrast image of a step edge without a GNR. Scale bar, 0.5 μm. (**e**) Phase shift profiles taken from images (**c**) (red dashed line) and (**d**) (yellow dashed line). (**f**) Cross-sectional phase-contrast TEM image of a bilayer GNR that extends along the surface terrace (growth at 1,450 °C for 15 min followed by air annealing). Scale bar, 20 nm. (**g**) A close-up view of the same region revealing the existence of few-layer graphene at the step facet. Scale bar, 5 nm.

Figure 2 illustrates a collection of atomic force microscopy (AFM) and transmission electron microscopy (TEM) results, obtained for bilayer GNRs prepared at different growth temperatures for 15 min, followed by an annealing in air at 600 °C for 40 min. The results are representative of the effect occurring at several step edges at the sample surface. Figure 2a shows an AFM phase-contrast image of a sample prepared at 1,500 °C. It can be clearly seen that μm-long nanoribbons are located exclusively near to the surface step edges, as verified by comparing Fig. 2a (phase-contrast) with Fig. 2b (height profile of the same region). The average width of the GNRs in this case is 320 nm. The inset of Fig. 2a is a close-up view of a single GNR prepared at this condition. Narrower ribbons could be synthesized by lowering the temperature. Indeed, ribbons with width ranging from about 30 to 320 nm can be fabricated on top of the BL by varying the growth temperature from 1,410 to 1,500 °C for 15 min (the variation of the GNR width could also be obtained by varying the growth time—see Supplementary Fig. 1). An example is given in Fig. 2c, which shows a close-up view of a single bilayer GNR prepared at 1,470 °C. In this case, the AFM image reveals an average width of about 200 nm. Note, however, that the step edge alone induces a phase contrast (of about 50 nm in width) due to the height variation, as can be observed in the image in Fig. 2d, and in the profiles shown in Fig. 2e. Therefore, in this specific case the bilayer GNR formed on the upper terrace is in fact about 150 nm wide.

The cross-sectional TEM image in Fig. 2f reveals that for a sample prepared at 1,450 °C the GNR is narrower: it extends about 70 nm over the upper terrace. It also confirms the isolated nature of the bilayer GNR, and gives more detailed information about the nature of the graphene grown on the step facet. The surfaces of the lower terrace as well as of the upper terrace next to the GNR are free of any graphene or BL. Surface oxidation is also not detected in these regions. These findings are in agreement with the illustration in Fig. 1d. A magnified image (Fig. 2g; see also Supplementary Fig. 2) shows that the number of graphene layers at the step facet differs from that present at the upper terrace. Contrast analysis (not shown) also confirms that while the portion grown at the upper terrace is essentially bilayer graphene, about six graphene layers are formed at the [1–10*n*] facet. The discontinuity and even appearance of three layers (at the upper terrace) in the high-resolution GNR image in Fig. 2g is probably related to damage due to the TEM process (see Supplementary Fig. 2). The formation of few-layer graphene at the non-polar step facets during the nucleation of epitaxial graphene has been modelled by Ming and Zangwill^26^ and experimentally observed by Robinson *et al.*^29^ On the basis of these works, it is reasonable to state that the dimensions of the facet (width and height) will determine the lateral extension of few-layer region within the GNR as well as the numbers of graphene layers formed there. Norimatsu and Kusunoki^18^ have shown that, if graphene nucleates at step facets with a sufficiently low height (∼2 unit cells of 6H-SiC, that is, 3 nm, or less), the same number of layers growing from the nano-facet will propagate over the upper terrace. We could obtain similar results (see Supplementary Fig. 3). Thus, the use of a SiC starting surface offering well-defined and low step facets (obtained for instance by pre-patterning^13^) will allow for the mitigation of the ‘parasitic' growth of few-layered graphene during the preparation of bilayer GNRs. Interestingly, the average distance between the bottom graphene layer and the SiC surface was found to be about 0.6–0.8 nm, which is much larger than the value reported for the interlayer distance between the BL and SiC (around 0.25 nm)^30^,^31^. This is likely related to the creation of an amorphous oxide interface between the GNR and the SiC surface, a direct product of oxygen intercalation^27^,^28^. This allows for the decoupling of the BL from the substrate, giving the GNR a quasi-free-standing nature. Note that the BL decoupling as described above is expected to take place only at the (0001) upper terrace where the bilayer GNR is formed. This is because graphene grown on SiC non-polar planes and facets are naturally detached from the substrate due to the lack of BL, as demonstrated by Nicotra *et al.*^30^ and Ostler *et al.*^32^ Therefore, in the present case it is plausible to assume that the air annealing will only change the type of interface at the step facet due to its oxidation.

### Spectroscopic characterization

**Figure 3:**
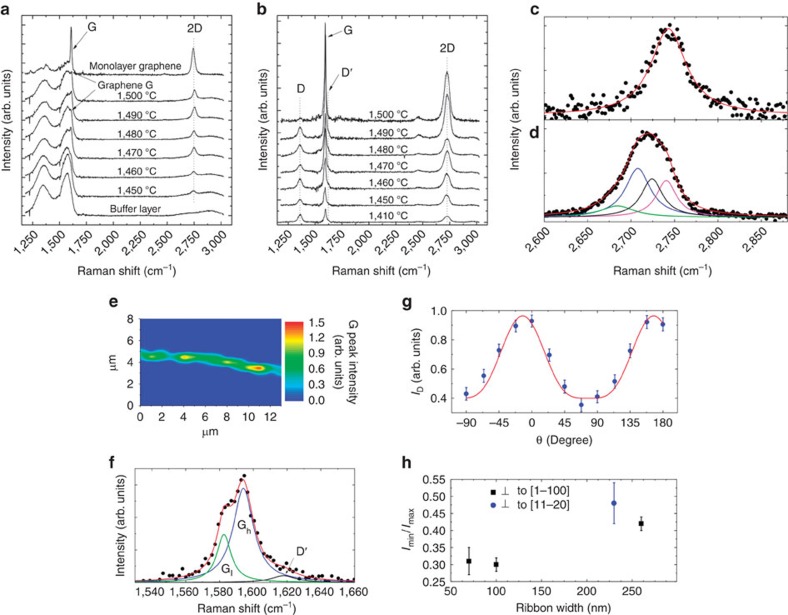
Raman results for monolayer and bilayer GNRs on SiC(0001). (**a**) Raman spectra of pristine monolayer GNRs (on top of the BL) grown on SiC(0001) for 15 min at different temperatures. Spectra of a bare BL (bottom spectrum) as well as from a monolayer graphene (on top of the BL, top spectrum) are plotted for comparison. (**b**) Raman spectra of quasi-free-standing bilayer GNRs created after thermally treating monolayer GNRs in air. (**c**,**d**) Raman 2D peaks measured for a GNR grown on a SiC surface step edge at 1,450 °C for 15 min (**c**) before and (**d**) after annealing in air. (**e**) Raman mapping (8 × 13 μm^2^) of the G peak intensity taken from a quasi-free-standing bilayer GNR synthesized at 1,470 °C for 15 min followed by air annealing. (**f**) Splitting of the Raman G peak into two components for the same bilayer GNR. (**g**) Dependence of the D peak intensity (*I*_D_) on the angle between the polarization of the linearly polarized laser and the GNR edge for a bilayer GNR prepared on surface steps perpendicular to the [1–100] direction (growth at 1,490 °C for 15 min followed by air annealing). The solid red line represents the best fitting obtained using the function *I*_min_+(*I*_max_–*I*_min_)cos^4^(*θ*) (ref. 42). (**h**) Dependence of the *I*_min_/*I*_max_ ratio on the ribbon width. The Raman spectra were recorded with spatial resolution of about 1 μm. Error bars in (**g**) and (**h**) represent s.d.

Raman spectra taken at the step edge regions of non-annealed GNR samples (see Fig. 1c) grown for 15 min at different temperatures are shown in Fig. 3a (results for different growth times are shown in Supplementary Fig. 1). For the sake of comparison, typical spectra recorded at terraces of a SiC(0001) surface fully covered either with a monolayer graphene (on top of the BL—not illustrated in Fig. 1) or only with the BL (see Fig. 1b) are depicted at the top and bottom of the plot, respectively. In the case of monolayer graphene, the spectrum contains the typical graphene-related peaks (G and 2D)^33^, as well as the contribution related to the underlying BL (broad bands between 1,250 and 1,650 cm^−1^—the most prominent features observed in the spectrum of the BL-covered sample)^34^. For the GNR samples, this BL-related signal is detected only in measurements performed away from the step edges, that is, exclusively on surface terraces. For spectra recorded at the step edge regions, although the BL-related signal is still visible, the 2D peak is observed at around 2,740 cm^−1^. Its low intensity is due to the fact that only a small fraction of the total area illuminated by the laser spot (about 1 μm in diameter) corresponds to monolayer graphene. The remaining area is covered only by the bare BL. For every GNR with width ≥70 nm (width at the upper terrace), synthesized at temperatures ≥1,450 °C for 15 min, the 2D peak can be well fitted by a single Lorentzian, demonstrating that monolayer graphene is formed. For narrower GNRs (that is, prepared at *T*<1,450 °C and t<15 min), a reliable peak fitting could not be done as the signal intensity in those cases was too low. Hence, information about the narrow stripe or isolated patches of few-layer graphene built at the step facet (for example, number of layers) could not be obtained by this technique. Note that the G peak (∼1,590 cm^−1^) cannot be clearly distinguished in the spectra as they lie within the same spectral range as the BL-related signal. The same is valid for the D (∼1,370 cm^−1^), which might exist owing to the edges of the monolayer GNRs.

Results obtained for the air annealed samples are depicted in Fig. 3b. The remarkable differences between these spectra and the ones shown in Fig. 3a are the lack of BL-related features and thus the clear observance of the graphene-related D, G and 2D peaks, even in the case where a growth temperature of 1,410 °C was used (that is, bilayer GNRs narrower than 40 nm). This confirms that the BL, which was originally covering the terraces next to the GNRs (see Fig. 1c), was etched away on thermal treatment. Moreover, the absence of any BL-related signal also implies that the BL lying underneath the monolayer GNR (see Fig. 1c) also underwent transformation^27^,^34^. Nevertheless, it has not been etched away in this region, but rather chemically detached from the substrate (due to SiC surface oxidation) and converted into a graphene layer. This interpretation, which can be inferred from the TEM results, is corroborated by analysing the shape of the 2D peak. Figure 3c,d shows a comparison between the 2D peak recorded for a GNR before (Fig. 3c) and after (Fig. 3d) annealing in air. As aforementioned, the 2D peak of the pristine sample (Fig. 3c) is well fitted by a single Lorentzian, which characterizes a monolayer graphene. In contrast, after air annealing it cannot be fitted in the same way, but it is well described by four Lorentzians (Fig. 3d), which is a typical characteristic of AB-stacked bilayer graphene^33^,^35^,^36^. Here, as in the case of the monolayer GNRs, the few-layer graphene area built solely at the step facet (see Fig. 2g) does not significantly contribute to the Raman spectrum owing to its small relative area and to the non-perpendicular incidence of the laser beam at this region. The red shift of the 2D peak after thermal annealing is a consequence of a change in the strain and doping levels promoted by the formation of an oxide interface^27^. A Raman mapping of the G peak intensity, taken around one step edge from the sample grown at 1,470 °C for 15 min, is shown in Fig. 3e. The measurements, which were taken after the thermal annealing, also serve to prove the existence of an isolated and laterally continuous GNR at a step edge region, as well as the absence of any graphene- or BL-related Raman features outside this area. The width of the GNR displayed in the mapping does not correspond to its real dimension, as the spatial resolution of the Raman set-up is about 1 μm. The real width is, in this case, ∼200 nm. Interestingly, as depicted in Fig. 3f, a G peak-splitting into two components, one at ∼1,584 cm^−1^ (called G_l_) and the other at ∼1,591 cm^−1^ (called G_h_), is measured. Such splitting probably occurs due to the existence of different doping levels between the two graphene layers, which leads to the breaking of the inversion-symmetry, resulting in the activation of the antisymmetric mode E_u_ (refs 37, 38, 39) in addition to the E_2g_ one. A similar effect has been observed for a large area quasi-free-standing bilayer graphene developed using the same annealing process^27^. It is also possible to visualize in Fig. 3f the D' peak that, as the D one, is related to the edges of the bilayer GNRs.

The intriguing selective removal of the BL formed on surface terraces might have its origin on the different atomic corrugation that this layer offers when it is bare or covered by a monolayer graphene. In a recent work, de Lima *et al.*^40^ showed that atoms of the sublattice A of a bare BL are in average 0.36 Å closer to the SiC surface than the ones from the sublattice B. This difference amounts to only 0.09 Å when the BL is covered by a monolayer graphene. Such an effect will result in a higher pyramidalization of the sp^2^-hybridized C atoms for the bare BL case. A direct consequence of this, as shown by Sclauzero and Pasquarello^41^, is the enhancement of their chemical reactivity. Therefore, in the present case, the bare BL existing on terraces is able to strongly react with oxygen-containing species during air annealing. Carbon is removed from the SiC surface terraces probably due to the formation of volatile species such as carbon monoxide and hydrocarbons. The same effect does not take place at the step edge regions where the GNRs grow. There, the oxygen precursors react preferentially with the SiC surface, as the BL is underneath a graphene layer and hence possesses a low reactivity and thus a higher stability.

Finally, Raman spectroscopy was also employed to investigate the edges of the fabricated GNRs. The D peak intensity (*I*_D_) has shown to be given by *I*_min_+(*I*_max_–*I*_min_)cos^4^(*θ*), where *θ* is the angle between the polarization of the linearly polarized photons and the ribbon edge (see Fig. 3g)^42^. Moreover, the D peak intensity never vanishes, and the ratio *I*_min_/*I*_max_ between its minimum value *I*_min_ and its maximum *I*_max_ was found to lie between 0.30 and 0.48. For samples whose steps are perpendicular to the [1–100] direction, this ratio varied between 0.30 and 0.42, which is characteristic of graphene edges containing long armchair segments^42^. Indeed, in this case the armchair-rich termination is expected due the epitaxial relation between epitaxial graphene and the SiC substrate^43^,^44^,^45^. Interestingly, *I*_min_/*I*_max_ increases with the ribbons width, indicating that the degree of disorder at the GNR edges increases as the graphene layer propagates along the terrace (Fig. 3h). In other words, narrower bilayer GNRs offer a more ordered edge termination. For bilayer GNRs prepared at steps perpendicular to the [11–20] direction, a larger value of *I*_min_/*I*_max_, 0.48, was found. Although the Raman analysis is not conclusive in confirming the edge chirality (due to the large error bar, as can be seen in Fig. 3h), this result suggests that in this case the GNRs are indeed preferentially zigzag-terminated^46^, a direct consequence of the epitaxial relation between graphene and SiC. Importantly, the Raman and AFM analyses shows that in general the GNR growth behaviour (as illustrated in Fig. 1) is very similar for both step directions, including the growth rate and the number of carbon layers. The same is valid for the bilayer production on air annealing. Hence, the edge type that the bilayer GNR will offer can in principle be pre-determined by choosing the appropriate miscut direction of the SiC substrate.

## Discussion

We have shown the direct synthesis of quasi-free-standing bilayer GNRs on SiC(0001) surfaces. By controlling the layered growth of graphene on SiC(0001) stepped surfaces, monolayer GNRs of different widths (ranging from about 30 to 320 nm) were fabricated on top of the BL by varying the growth conditions. The GNRs are formed exclusively over step edge regions, while the BL is extended over the terraces. Strikingly, by annealing the samples in air, the uncovered BL is selectively etched, while the monolayer GNRs are converted into quasi-free-standing bilayer GNRs. The generalization of this GNR synthesis method to pre-patterned SiC surfaces^13^ is very promising for achieving the precise fabrication of bilayer graphene nanostructures over large areas for different applications in nanoelectronics.

## Methods

### Sample preparation

Epitaxial monolayer GNRs were grown on 6H-SiC(0001) substrates (0.5 × 1 cm^2^) cut from a nominally on-axis 2 inch semi-insulating wafer polished on the (0001) face. The samples were chemically cleaned in n-butyl-acetate, acetone and methanol. The following processing steps, namely the H-etching and GNR growth, were both performed in a furnace equipped with an induction heating system. The H-etching treatment was carried out at 1,550 °C for 15 min in a forming gas atmosphere (95 at.% Ar and 5 at.% H) of 900 mbar and a flow rate of 500 s.c.c.m. The epitaxial monolayer GNRs were prepared at the 1,410–1,500 °C temperature range for 5–15 min in a 900 mbar Ar atmosphere with a flow rate of 500 s.c.c.m.

The choice for growth duration of 15 min was not arbitrary. The synthesis of a complete monolayer graphene on Si(0001), using our experimental set-up, is achieved after a 15 min long process at a temperature of 1,600 °C^19^,^27^. Since epitaxial graphene on SiC(0001) grows layer-by-layer from the steps edges, there were two ways of confining the growth process to the region near to the step edge (to prepare GNRs): (1) by changing the temperature (and keeping a 15 min growth time); (2) by changing the growth time (and keeping the growth temperature constant).

Oxygen intercalation/etching was accomplished by thermally treating the samples for 40 min at 600 °C in air with a preceding heating ramp of 50 °C min^−1^.

### Raman spectroscopy

Raman measurements were performed before and after oxygen intercalation using the 482.5 nm-line of a Kr^+^ ion laser for excitation. The spatial resolution (∼1 μm) allowed performing measurements of a single step edge, since the typical terrace width is larger than 1 μm, as determined by AFM. Spectra of all samples were obtained under the same experimental conditions (integration time and incident laser power) to allow a direct comparison of the absolute intensity between them. Furthermore, the Raman signal of a bare SiC substrate was subtracted from all original data. Raman mapping was taken from an 8 × 13 μm^2^ area around one step edge. Polarized Raman measurements were carried out by coupling a half-wave plate to the microscopy optics, which allows rotating the polarization of the incident and scattered light, but keeping them parallel to each other. An analyser was employed to ensure that the dependence of the spectrometer efficiency on the light polarization does not affect the experiments. Measurements were performed as a function of the angle between the polarization of the linearly polarized laser and the longitudinal direction of the ribbons. In all cases the absolute position of the Raman peaks was corrected by using the emission lines of a Neon lamp.

### Atomic force microscopy

AFM measurements were taken using the tapping mode to obtain the height and phase contrast images. The choice for phase contrast is justified as this mode gives information about regions of the SiC surface covered by different numbers of graphene layers.

### Transmission electron microscopy

The TEM samples used for the analysis were prepared in cross-sectional geometry using standard procedure of mechanical grinding and dimpling. Afterwards they have been thinned by argon ion beam milling with an energy of 4 keV under an incident angle of 4° using a Gatan precision ion polishing system. The TEM data were collected in a JEM-2100F operated at 80 kV equipped with a field emission gun. Additional TEM images were taken with a JEM-3010 operated at 200 kV.

## Additional information

**How to cite this article:** Oliveira Jr., M.H. *et al.* Synthesis of quasi-free-standing bilayer graphene nanoribbons on SiC surfaces. *Nat. Commun.* 6:7632 doi: 10.1038/ncomms8632 (2015).

## Supplementary Material

Supplementary Information Supplementary Figures 1-3
