# Supplementary material for: Synthesis of quasi-free-standing bilayer graphene nanoribbons on SiC surfaces
Source: Nat Commun. 2015 Jul 9;6:7632. doi: 10.1038/ncomms8632 (PMC4510648; doi:10.1038/ncomms8632)
Supplement: Supplementary Information — Supplementary Figures 1-3 [file ncomms8632-s1.pdf]

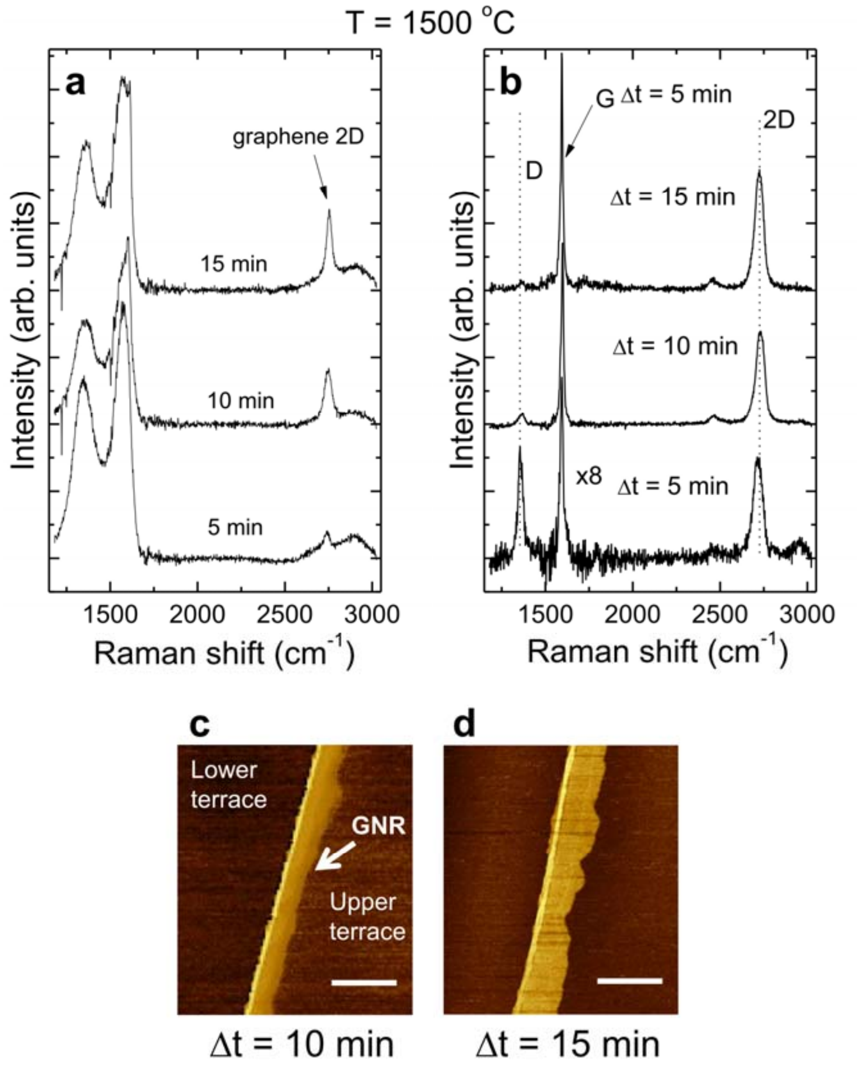

Supplementary Figure 1 – Raman spectra and AFM images for monolayer and bilayer GNRs on SiC(0001).

(a) Raman spectra of pristine monolayer GNRs (on top of the BL) grown on SiC(0001) at 1500 °C for 5, 10, and 15 min. **(b)** Raman spectra of quasi-free-standing bilayer GNRs created after thermally treating monolayer GNRs in air. **(c)** AFM phase contrast image taken from a single bilayer GNR (growth at 1500 ºC for 10 min followed by air annealing). Scale bar, 0.5 μm. **(d)** AFM phase contrast image taken from a single bilayer GNR (grown at 1500 ºC for 15 min followed by air annealing - note that a modified version of the same image is shown as an inset in Fig. 2a in the manuscript). Scale bar, 0.5 μm. It was not possible obtain a proper image of GNRs prepared for 5 min, since their related phase-contrast overlaps with that induced by the step edge.

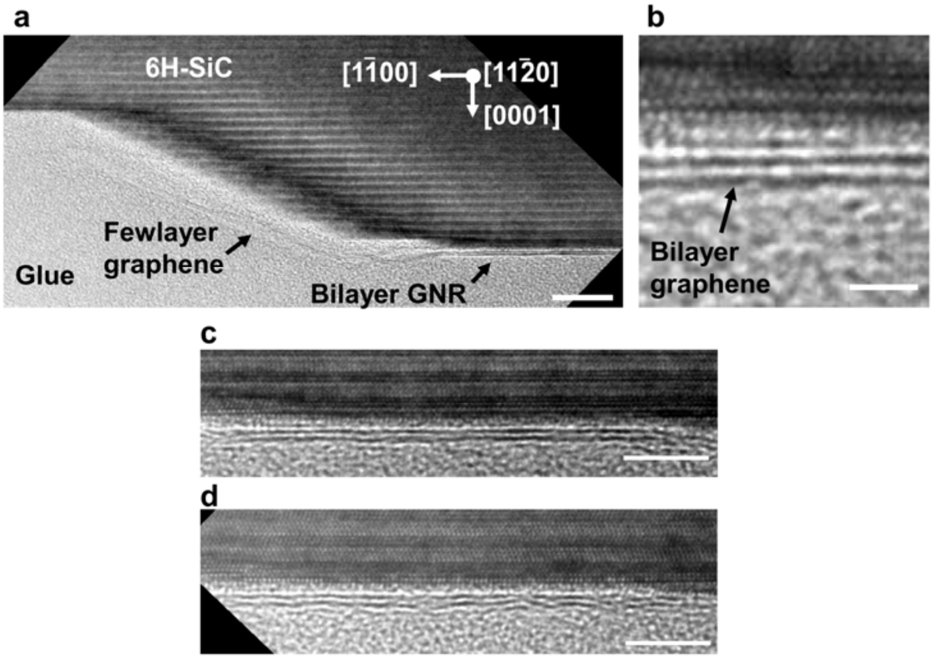

Supplementary Figure 2 – TEM images for bilayer GNRs on SiC(0001).
(a) Cross sectional phase contrast TEM image of a bilayer GNR which extends along the surface terrace (width of about 30 nm). Scale bar, 10 nm. **(b)** Close up view of the upper terrace revealing the existence of bilayer graphene. Scale bar, 2 nm. **(c)** Phase contrast TEM image of bilayer graphene obtained after a short period of beam exposure. Scale bar, 5 nm. **(d)** Image of the same region after several minutes of beam exposure. The bilayer graphene appears clearly damaged after being exposed to the electron beam. Scale bar, 5 nm.

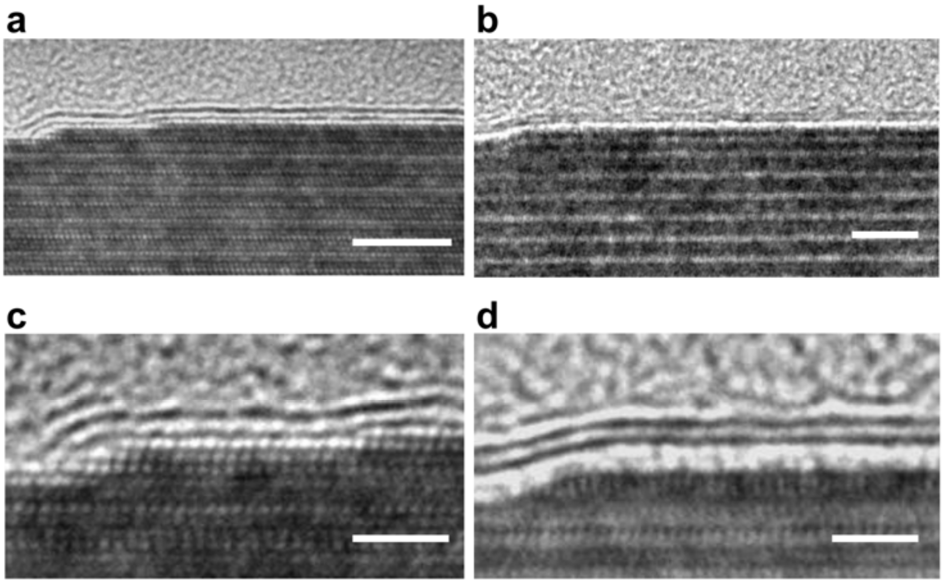

Supplementary Figure 3 – TEM images of GNRs grown from a low-height step edge.
(a) Cross sectional phase contrast TEM image of a monolayer GNR (see Fig. 1*c* in the article) grown from a low-height step edge at 1450 °C for 15 min. Scale bar, 5 nm. **(b)** Cross sectional phase contrast TEM image of a bilayer GNR (see Fig. 1*d* in the article) obtained after air annealing (for the same sample shown in (a), not the same step edge). Scale bar, 5 nm. **(c,d)** Close up views of the step edge area for the images shown in (a) and (b), respectively. The same number of layers is seen at the edge and upper terrace. Scale bars, 2 nm.
